# Survey on Different Samsung with Nokia Smart Mobile Phones in the Specific Absorption Rate Electrical Field of Head 

**DOI:** 10.5539/gjhs.v8n9p251

**Published:** 2016-01-31

**Authors:** Yadolah Fakhri, Azim Alinejad, Hassan Keramati, Abotaleb Bay, Moayed Avazpour, Yahya Zandsalimi, Bigard Moradi, Leila Rasouli Amirhajeloo, Maryam Mirzaei

**Affiliations:** 1 Social Determinants in Health Promotion Research Center, Hormozgan University of Medical Sciences, Bandar Abbas, Iran; 2 Department of Environmental Health Engineering, School of Public Health, Shahid Beheshti University of Medical Sciences, Tehran, Iran; 3 Department of Environmental Health Engineering, School of Public Health, Semnan University of Medical Sciences, Semnan, Iran; 4 Environmental Health Research Center, Golstan University of Medical Sciences, Golstan, Iran; 5 Department of Environmental Health Engineering, School of Public Health, Ilam University of Medical Sciences, Ilam, Iran; 6 Environmental Health Research Center, Kurdistan University of Medical Sciences, Sanandaj, Iran; 7 Department of Health Public, Kermanshah University of Medical Sciences, Kermanshah, Iran; 8 Department of Environmental Health Engineering, School of Public Health, Qom University of Medical Sciences, Qom, Iran; 9 Jahrom University of Medical Sciences, Jahrom, Iran

**Keywords:** smart mobile phones, Samsung, Nokia, specific absorption rate

## Abstract

The use of smart phones is increasing in the world. This excessive use, especially in the last two decades, has created too much concern on the effects of emitted electromagnetic fields and specific absorption rate on human health. In this descriptive-analytical study of the electric field resulting from smart phones of Samsung and Nokia by portable measuring device, electromagnetic field, Model HI-3603-VDT/VLF, were measured. Then, head absorption rate was calculated in these two mobiles by ICNIRP equation. Finally, the comparison of specific absorption rate, especially between Samsung and Nokia smart phones, was conducted by T-Test statistics analysis. The mean of electric field for Samsung and Nokia smart mobile phones was obtained 1.8 ±0.19 v/m and 2.23±0.39 v/m, respectively, while the range of the electric field was obtained as 1.56-2.21 v/m and 1.69-2.89 v/m for them, respectively. The mean of specific absorption rate in Samsung and Nokia was obtained 0.002 ± 0.0005 W/Kg and 0.0041±0.0013 W/Kg at the frequency of 900 MHz and 0.004±0.001 W/Kg and 0.0062±0.0002 W/Kg at the frequency of 1800 MHz respectively. The ratio of mean electronic field to guidance in the Samsung mobile phone at the frequency of 900 MHz and 1800 MHz was 4.36% and 3.34%, while was 5.62% and 4.31% in the Nokia mobile phone, respectively. The ratio of mean head specific absorption rate in smart mobile phones of Samsung and Nokia in the guidance level at the frequency of 900 was 0.15% and 0.25%, respectively, while was 0.23% and 0.38% at the frequency of 1800 MHz, respectively. The rate of specific absorption of Nokia smart mobile phones at the frequencies of 900 and 1800 MHz was significantly higher than Samsung (p value <0.05). Hence, we can say that in a fixed period, health risks of Nokia smart phones is higher than Samsung smart mobile phone.

## 1. Introduction

**Figure 1:**
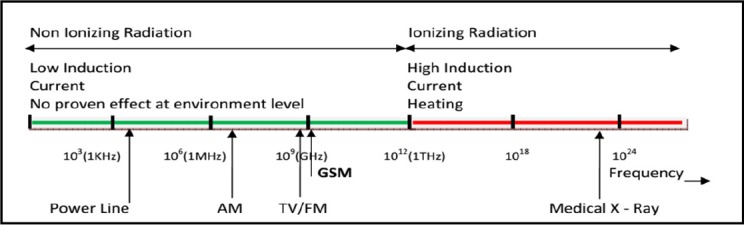
The frequency of Ionizing and non-ionizing rays such as mobile, TV, power lines, etc

Nowadays, exposure to electromagnetic fields emitted by mobile phone, telecommunication antennas, television, laptop, tablet, high voltage power cables, etc is inevitable (Joseph, Frei, Roösli, Thuróczy, & Gajsek, 2010; Guidotti, From, & Martinez, 2007). Using a mobile phone was launched in 1983 and today many people are using this tool (Bortkiewicz, Gadzicka, Szymczak, & Zmyślony, 2012). For example, in 2011, 129.86 million of 140 million population of Japan used smart mobile phones, and 91% of population of the United States used smart mobile phones and 91% of population of Great Britain used smart mobile phones (Nakatani-Enomoto, Furubayashi, Ushiyama, Groiss, & Ueshima, 2013; Gajšek, Ravazzani, Wiart, Grellier, & Samaras, 2015; Saltos, Smith, Schreiber, Lichenstein, & Lichenstein, 2015). Additionally, the ownership of mobile phone ownership was increased from 12% in 1999 to 76% in 2009. According to the number of assigned SIM cards, Iran has the penetration rate of 130% of mobile phone (Mehrnews, 2013). Although, multiple global and national guidelines have been developed since early 1950s concerning the exposure to electromagnetic field, concerns about the unknown effects of this field, even at lower levels, still is growing (Vecchia, Matthes, Ziegelberger, Lin & Saunders, 2009). This overuse, especially in the last two decades, caused a lot of concern on the effects of EMFs emitted by smart phones on human health (Hauri, Spycher, Huss, Zimmermann & Grotzer, 2014; Nath & Mukherjee, 2015; Pourlis, 2009). Radiations are divided into Ionizing and non-ionizing categories (Figure 1) (Morgan, & Sowa, 2015). Many reports show that exposure to non-ionizing radiations, such as EMFs can cause effects such as headaches, poor concentration and memory, fatigue, drowsiness and anxiety in humans (Arnetz, Åkerstedt, Hillert, Lowden, & Kuster, 2007). Electromagnetic has a adverse effects on the reproductive system such as infertility (Kesari, Kumar, & Behari, 2010). Additionally, EMFs could also have damaging effects in other creatures, for example, if exposed to EMFs of mobile (900 MHz), the cells of earthworm of fetida Eisenia will be damaged (Tkalec, Štambuk, Šrut, Malarić, & Klobučar, 2013) and it causes reproductive disorders in birds and mice (Balmori, 2009). The World Health Organization has categorized EMFs emitted by cell phones as 2B class (possibly carcinogenic) in terms of carcinogenesis in class (WHO, 2011).

Frequency of communications networks in Iran is 900 and 1800 MHz (Figure 1). Therefore, 53.8 m/v and 41.25 m/v have been considered as guidelines for public exposure (ICNRP, 2009) Studies have shown that at frequencies greater than 100 MHz such as mobile frequencies, human exposure assessment is very important by calculation of SAR (specific absorption rate) (Ahma, Ibrani, & Hamiti, 2010). Diameter of Seminiferous Tubules in mice after one month of exposure to electromagnetic field was reduced in the absorption of 0.141 W/kg (Sarookhani, Rezaei, Safari, Zaroushani & Ziaeiha, 2011). International Commission on Non-Ionizing Radiation Protection for the Specific Absorption electromagnetic field recommended 2 W/Kg per 10 g, and Institute of Electrical and Electronics Engineers and the World Health Organization recommended 1.6 W/Kg per 10 g (ICNRP, 2010). In recent years, many studies have focused on impact of electromagnetic fields on health (Stein, Hänninen, Huttunen, Ahonen, & Ekman, 2015), clinical disease (Fujii, 2015) and behavioral effects (Thomas, Heinrich, von Kries, & Radon, 2010). However, less attention has been paid on the specific absorption rate of electromagnetic field of smart mobile phones. Therefore, in this study we have attempted to compare and evaluate the difference in the rate of specific absorption of electric field in the Samsung and Nokia smart mobile phones.

## 2. Mterials & Methods

### 2.1 Measuring the Electronic Field

**Figure 2:**
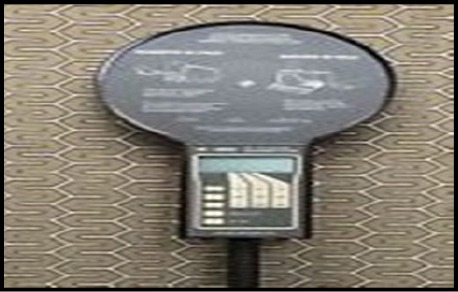
The portable device to measure electromagnetic field HI-3603 VDT/VLF Model

This descriptive-analytical study was conducted in October 2014 when two smart phone brands were selected among the world famous brands called as as Samsung and Nokia, firstly. Five models of each brand were selected to measure the electric field by EMFs survey meter model HI 3603 device (Figure 2). All five models of each brand were named alphabetically from A to E. Before start to measure, the electric field that can be caused by other electrical equipment such as telecommunication antenna, substation, television, were measured. As humans stick the mobile phone to their ear when talking, therefore, the measurement of field was conducted from 5 mm distance. Measurement was done in conditions of without vibration and without an Internet connection and only in alarm mode for all mobile phone models. Finally, electric field was calculated for each model of mobile phone according to Equation 1;

EF(MobilePhone)=EF(Measured)-EF (background) (1)

### 2.2 Calculation of the Specific Absorption Rate

To calculate the specific absorption rate of the electric field, the Equation 2 was used that is developed by International Commission on Non-ionizing Radiation Protection (ICNIRP, 2009);


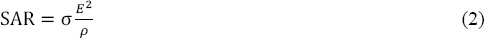


In this equation, SAR is specific absorption rate of electric field (W/kg), σ is conductivity of head tissue (Ω^-1^m^-1^) in which 900 MHz and 1800 MHz is 0.7665 Ω^-1^m^-1^ and 1.1531 Ω^-1^m^-1^, respectively and ρ is mass density of head (Kgm^-3^) which is 1030 Kgm^-3^ both in 900 MHz and 1800 MHz (ICNIRP, 2009).

### 2.3 Statistical Analysis

After determining the normal distribution of data, t-test was used for statistical analysis in the spss 16. To compare the specific absorption rate of electric field of samsung and nokia, independent sample t-test was used, to compare the different models with each other at frequencies of 900 and 1800 MHz, one way anova test was used, and to compare the electric field and the specific absorption rate with guidelines, one sample t test was used. P value <0.05 was chosen as the significance level (α = 5%).

## 3. Results

The mean (M±SD) of electric field of samsung and nokia smart mobile phones (Equation 1) was respectively 1.8± 0.19 v/m and 2.32±0.39 v/m. The electric field rang in samsung and nokia is respectively 2.21-1.56 v/m and 1.69- 2.89 (Table 1). Additionally, the mean of background electric field in the measurement time was 0.19 v/m and 0.39 v/m for samsung and nokia, respectively.

**Table 1:** The electric field in the 5 models of smart mobile phones of Samsung and Nokia (v/m)

Model	Samsung (Measured)	Background	Samsung (Mobile Phone)	Nokia (Measured)	Background	Nokia (Mobile Phone)
	1.75	0.19	1.56	2.1	0.21	1.89
	1.8	0.19	1.61	2.1	0.21	1.89
	2.4	0.19	2.21	2.1	0.21	1.89
	2.2	0.19	2.01	2.2	0.21	1.99
**A**	1.95	0.19	1.76	2.2	0.21	1.99
	2	0.19	1.81	2.2	0.21	1.99
	2	0.19	1.81	2.7	0.21	2.49
	1.9	0.19	1.71	2.6	0.21	2.39
	1.8	0.19	1.61	2.6	0.21	2.39
**B**	1.5	0.19	1.31	2.6	0.21	2.39
	1.9	0.19	1.71	2.6	0.21	2.39
	1.8	0.19	1.61	2.5	0.21	2.29
	2.2	0.19	2.01	2.9	0.21	2.69
	1.95	0.19	1.76	2.9	0.21	2.69
	2	0.19	1.81	3.1	0.21	2.89
	1.9	0.19	1.71	3	0.21	2.79
**C**	1.9	0.19	1.71	3	0.21	2.79
	1.8	0.19	1.61	3	0.21	2.79
	2	0.19	1.81	2.7	0.21	2.49
	2.2	0.19	2.01	2.9	0.21	2.69
	2.2	0.19	2.01	2.9	0.21	2.69
**D**	2.2	0.19	2.01	2.9	0.21	2.69
	2.2	0.19	2.01	2.9	0.21	2.69
	2.2	0.19	2.01	2.9	0.21	2.69
	2.1	0.19	1.91	1.9	0.21	1.69
	2.1	0.19	1.91	2	0.21	1.79
	1.9	0.19	1.71	2	0.21	1.79
	2	0.19	1.81	2.1	0.21	1.89
**E**	1.9	0.19	1.71	2.2	0.21	1.99
	1.9	0.19	1.71	2.1	0.21	1.89
**Mean**			**1.80**			**2.32**
**Standard Deviation**			**0.19**			**0.39**

The mean (M±SD) of specific absorption rate in Samsung and Nokia was 0.0024±0.0005 W/Kg and 0.0041±0.0013 W/Kg at the frequency of 900 W/Kg, while it was 0.004±0.001 W/Kg and 0.0062±0.002 at the frequency of 1800 MHz, respectively (Table 2). At the frequency of 900 MHz, the mean of specific absorption rate in model A of Samsung is 0.0025 W/Kg, in the model B of Samsung is 0.0020 W/Kg, in the model C of Samsung is 0.0023 W/Kg, in the model D of Samsung is 0.0029 W/Kg, and in the E model of Samsung is 0.0024 W/Kg. While, in the A model of Nokia, it is 0.0028 W/Kg, in the B model of Nokia is 0.0042 W/Kg, in the model C of Nokia is 0.0057 W/Kg, in the model D of Nokia is 0.0053 W/Kg, and in the E model of Nokia is 0.0025 W/Kg (Table 2). In the Samsung, the mean of specific absorption rate frequency is 0.0038 W/Kg in the A model, 0.0030 W/Kg in the B model, 0.0035 W/Kg in the model C, 0.0044 W/Kg in the D model, and 0.0036 W/Kg in the E model, at the frequency of 1800 MHz. In the Nokia case, it was 0.0042 W/Kg in the A model, 0.0064 W/Kg in the B model, 0.0086 W/Kg in the model C, 0.0079 W/Kg in the E model, and 0.0038 W/Kg in the E model (Table 2).

**Table 2:** Specific absorption rate by the electric field in 5 models of Samsung and Nokia smart mobile phones (W/Kg)

	SAR			

	900 MHz			1800 MHz
	
Model	Samsung	Nokia	Samsung	Nokia
	0.0018	0.0027	0.0027	0.0040
A	0.0019	0.0027	0.0029	0.0040
	0.0036	0.0027	0.0055	0.0040
	0.0030	0.0029	0.0045	0.0044
	0.0023	0.0029	0.0035	0.0044
	0.0024	0.0029	0.0037	0.0044
Mean	0.0025	0.0028	0.0038	0.0042
	0.0024	0.0046	0.0037	0.0069
	0.0022	0.0042	0.0033	0.0064
	0.0019	0.0042	0.0029	0.0064
B	0.0013	0.0042	0.0019	0.0064
	0.0022	0.0042	0.0033	0.0064
	0.0019	0.0039	0.0029	0.0059
Mean	0.0020	0.0042	0.0030	0.0064
	0.0030	0.0054	0.0045	0.0081
	0.0023	0.0054	0.0035	0.0081
	0.0024	0.0062	0.0037	0.0094
C	0.0022	0.0058	0.0033	0.0087
	0.0022	0.0058	0.0033	0.0087
	0.0019	0.0058	0.0029	0.0087
Mean	0.0023	0.0057	0.0035	0.0086
	0.0024	0.0046	0.0037	0.0069
D	0.0030	0.0054	0.0045	0.0081
	0.0030	0.0054	0.0045	0.0081
	0.0030	0.0054	0.0045	0.0081
	0.0030	0.0054	0.0045	0.0081
	0.0030	0.0054	0.0045	0.0081
Mean	0.0029	0.0053	0.0044	0.0079
	0.0027	0.0021	0.0041	0.0032
D	0.0027	0.0024	0.0041	0.0036
	0.0022	0.0024	0.0033	0.0036
	0.0024	0.0027	0.0037	0.0040
	0.0022	0.0029	0.0033	0.0044
	0.0022	0.0027	0.0033	0.0040
Mean	0.0024	0.0025	0.0036	0.0038

Mean (Total)	0.0024	0.0041	0.0040	0.0062
SD	0.0005	0.0013	0.001	0.002

## 4. Discussion

**Figure 3:**
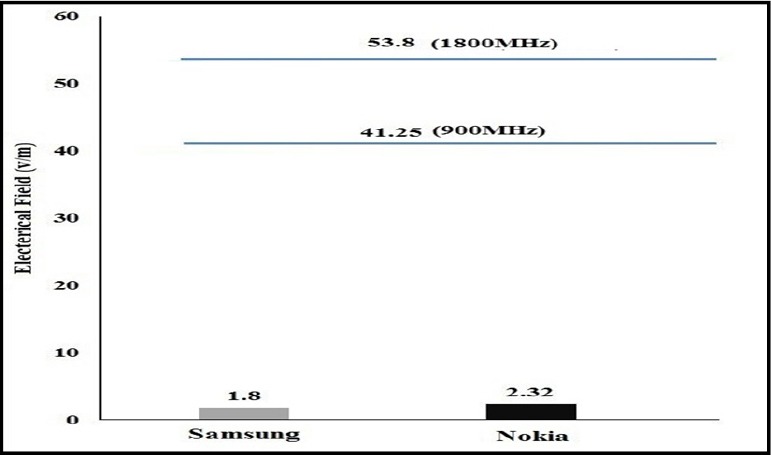
Comparing the mean of electric field of smart phones of Samsung and Nokia with the guidelines at the frequency of 900 MHz and 1800 MHz

The ratio of mean electric field of Samsung mobile phone in the guidance level at the field frequency of 900 MHz and 1800 MHz was 4.36% and 3.34%, while it was 5.62% and 4.31% in the Nokia mobile phone, respectively (Figure 3). Since the electric field is heavily dependent on the amperage, and the smart mobile phones have a lower amperage than many other electrical appliances, the electric field is lower in them (Yamaoka, Shinozaki, Maeda, Shimazaki, & Kato, 2004; Kumari, 2008). In the study conducted by Ghaffari et al, the electric field of smart phones was 1.78 m/v at a distance of 5 cm that was lower than the lower our study. The lower of electric field in our study is resulting from more distance, different brand of phone, Internet connection, phone life, phone mode (ring, vibrate or silent) (Kühn, Cabot, Christ, Capstick, & Kuster, 2009; Micheli, Delfini, Santoni, Volpini, & Marchetti, 2015).

**Figure 4:**
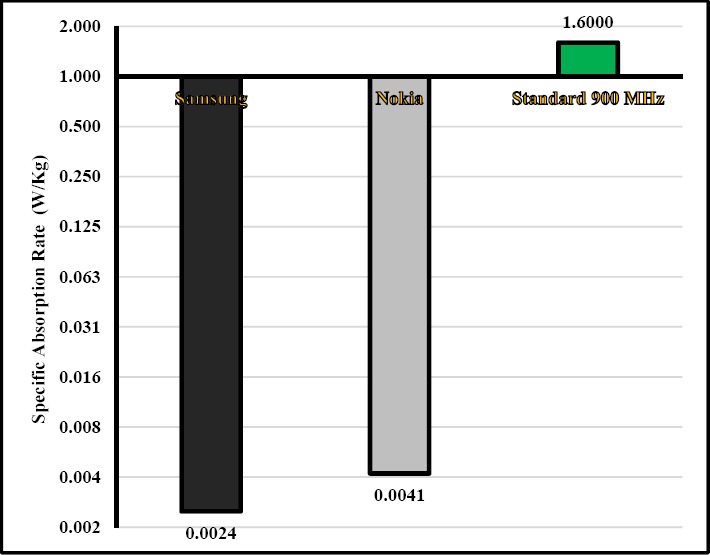
Comparison of mean of specific absorption rate in smart mobile phones of Samsung and Nokia with the guideline at the frequency of 900 MHz

**Figure 5:**
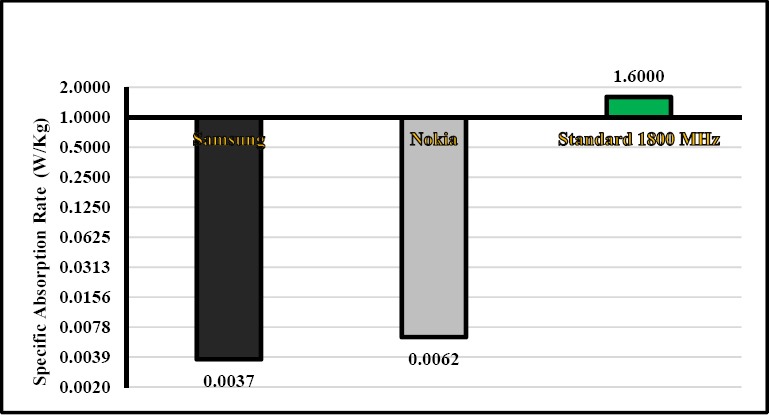
Comparison of mean of specific absorption rate in smart mobile phones of Samsung and Nokia with the guideline at the frequency of 1800 MHz

The ratio of mean head specific absorption rate to guidance in the smart mobile phones of Samsung and Nokia at the frequency of 900 was respectively 0.15% and 0.25%, while it was 0.23% and 0.38% at the frequency of 1800 MHz. As can be seen in Figures 4 and 5, as the electric field of these smart mobile phones was very low (Figure 3), the specific absorption rate is also very low (Figures 4 and 5). Since conductivty in 1800 MHz is high than 900 MHz (Equation 2), specific absorption rate is high. The head specific absorption rate is 1.57 W/kg at the distance of 1.01 mm in the study Naif. This specific absorption rate in the Naif is much more than our study (Naif, 2010). In a study Burdalo et al, the absorption rate for adults at a frequency of 900 and 1800 MHz was 0.02 W/Kg and 0.008 W/Kg, respectively that it is close to our study (Martinez-Burdalo, Martin, Anguiano, & Villar, 2004). Based on manufacturer of Nokia statement, the mean of SAR was 0.75±0.27 v/m in 116 models of Nokia mobile phone, which is much higher than our study and this difference is significant (Nokia, Sep 2015). Moreover, based on manufacturer of Samsung statement, the mean of SAR was 0.65±0.273 W/Kg in 96 models of Samsung mobile phone, which is much higher than our study and this difference is significant (Samsung, Sep 2015).

Specific absorption rate of smart mobile phones of samsung and nokia at the frequencies of 900 and 1800 MHz is significantly different (p value <0.05). This significant difference is due to the difference in the designing of two devices (see Table 3).

**Table 3:** Independent sample t test statistical analysis of specific absorption rate of smart mobile phones of samsung and nokia at two frequency of 900 MHz and 1800 MHz

	P value	Mean Difference	95% Confidence Interval of the Difference	

			Lower	Upper
900 MHz	<0.001	-0.0016	-0.0022	-0.0011
1800 MHz	<0.001	-0.0025	-0.0033	-0.0017

One way ANOVA statistical analysis showed that smart phones of A and B Model of Samsung were significantly different, B Model has significant difference with Models of A, D and E, C Model has significant difference with Model of D, Model D has significant difference with Models of B, C and E, and E model has significant difference with Models of B and E (P value <0.05). In the smart mobile phones of Nokia, Model A has significant difference with Models of B, C, and D, Model B has significant difference with Models of A, C, and D, there is significant difference between Model C and Model A and between Model E and Model B, Model D has significant difference with Models of A and E, and Model E has significant difference with Models of B, C, and E. As noted, these differences in various models are due to differences in electric equipment and design of smart mobile phones (Table 4). Since the specific absorption rate is multiplied in the constant numbers at the frequency of 1800 MHz, their difference is like the frequency of 900 MHz. In general, ANOVA statistical analysis showed that there is difference between 5 models of smart mobile phones of Nokia (P value= 0.021) and Samsung (P value= 0.016). Thus, we can say that there is a significant difference between the models of smart mobile phones of Samsung and Nokia in the specific absorption rate (p value <0.05).

**Table 4:** Statistical analysis of ANOVA for specific absorption of various models of samsung and nokia at 900 MHz

	Samsung		Nokia	
	
Model		p value		p value
A	B	0.043	B	0.012
	C	0.497	C	<0.001
	D	0.11	D	0.001
	E	0.683	E	0.308

B	A	0.043	A	0.012
	C	0.16	C	0.011
	D	0.001	D	0.282
	E	0.097	E	0.001

C	A	0.497	A	0.000
	B	0.16	B	0.011
	D	0.027	D	0.112
	E	0.785	E	<0.001

D	A	0.11	A	0.001
	B	0.001	B	0.282
	C	0.027	C	0.112
	E	0.049	E	<0.001

E	A	0.683	A	0.308
	B	0.097	B	0.001
	C	0.785	C	<0.001
	D	0.049	D	<0.001

## 5. Conclusions

The electric field mean and the specific absorption rate of head in the Samsung and Nokia smart mobile phones is much lower than guidance (p value <0.05). Specific absorption rate frequency in the 900 MHz is more than 1800 MHz. Nokia smart mobile phone models have significant difference with each other, and models of Samsung smart mobile phones have significant differences with each other in the specific absorption rate. Since the specific absorption rate of head electric field in the Nokia mobile phone is higher than Samsung (p value <0.05), it is recommended that Nokia smart mobile phones to be used with more caution.
